# Draft Genome Sequence of *Yarrowia lipolytica* Strain A-101 Isolated from Polluted Soil in Poland

**DOI:** 10.1128/genomeA.01094-16

**Published:** 2016-10-06

**Authors:** Hugo Devillers, François Brunel, Xymena Połomska, Véronique Sarilar, Zbigniew Lazar, Małgorzata Robak, Cécile Neuvéglise

**Affiliations:** a Micalis Institute, INRA, AgroParisTech, Université Paris-Saclay, Jouy-en-Josas, France; b Department of Biotechnology and Food Microbiology, Wrocław University of Environmental and Life Sciences, Chelmonskiego, Wrocław, Poland

## Abstract

*Yarrowia lipolytica* is an early diverging species of the *Saccharomycotina* subphylum, which is recognized as a valuable host for many biotechnological applications exploiting its oleaginous capacities. The 20.5-Mb genome of the Polish *Y. lipolytica* strain A-101 will greatly help decipher the genetic basis of the regulation of its lipid metabolism.

## GENOME ANNOUNCEMENT

*Yarrowia lipolytica* is one of the most extensively studied oleaginous yeasts used as a cell factory, which shows great capacity to produce a number of biotechnologically important metabolites, such as organic acids, enzymes, polyols, fatty acids, and aromas (1–7). Due to its ability to degrade organic compounds, including aliphatic and aromatic hydrocarbons, *Y. lipolytica* is also used in bioremediation and environment protection (8, 9).

Up to now, the whole-genome sequences of three *Y. lipolytica* strains are available, E150/CLIB122 (10), WSH-Z06 (BioProject PRJEB5051), and PO1f (11), as well as a draft of the W29 genome sequence (12). W29 and its derivative PO1f originate from France, whereas E150 derives from a cross between W29 and the American strain CBS6124-2. WSH-Z06 is a Chinese strain used for alpha-ketoglutaric acid production (13). Here, we present the genome sequence of strain A-101 isolated from polluted soil at a car wash at Wrocław, Poland (14). A-101 has been investigated for citrate biosynthesis from various substrates in different bioreactor systems (15, 16) and was used for soil bioremediation (17, 18). It was intensively mutated and genetically engineered to produce strains improved for citrate (19) and erythritol (16) biosynthesis.

A-101 DNA was sequenced with the Illumina HiSeq DNA sequencing platform (paired-end [PE] 2 × 100 bp), with a shotgun library of 280-bp inserts and a mate-pair library of 7.2 kb on average. The raw reads were trimmed with Trimmomatic version 0.32 (20) and cutadapt version 1.8.3 (21). The assembly was done using SOAP*denovo*2 version 2.04 (22), with a k-mer of 59, as estimated with kmergenie version 1.67 (23). Two successive runs of GapCloser from the Short Oligonucleotide Analysis Package (http://soap.genomics.org.cn/index.html) were used to close gaps, and manual curation was performed in overlapping regions. The current draft genome sequence is composed of 29 scaffolds larger than 5 kb (28 nuclear and a mitochondrial one), for a total size of nuclear DNA of 20,581,016 bp, with an *N*_50_ of 2,267,247 bp (4 scaffolds) and a G+C content of 49.03%. Coding sequence (CDS) prediction was essentially performed using the Amadea automatic annotation transfer software (24, 25), with E150 as the reference genome. A total of 6,576 putative CDS (including 15 alternative isoforms) were identified after manual curation. tRNA genes were determined using tRNAscan-SE version 1.4 (26) and 5S rRNA genes by similarity to E150. Transposable elements (TE) were manually annotated by similarity to yeast TE, including those of strain E150 (27). In addition to copies of Tyl6, Ylli, and Mutyl, a relic of Fotyl, and a single long terminal repeat (LTR) of Ylt1, a new full-length TE of the *Ty1/Copia* superfamily, named Tyl5, was identified with LTR corresponding to the previously known LTRyl1 (28).

By mapping the reads to the E150 genome using BWA version 0.7.10 (29) and analyzing them using Samtools version 1.2 (30), a total of 38,802 single nucleotide polymorphisms and 3,353 short indels were called in the A-101 genome sequence (nucleotide frequency of the reference (QS), <0.1; read depth (DP), ≥30), i.e., 2,048 nucleotide variations per Mb. Further comparison of this genome against other strains of *Y. lipolytica* and species of the *Yarrowia* clade will bring additional insights into gene functions and evolutionary events.

### Accession number(s).

This whole-genome shotgun analysis has been deposited at DDBJ/EMBL/GenBank under the accession no. FLLM00000000 (BioProject PRJEB14097 and scaffold sequences LT576309 to LT576337). The version described in this project is the first version, FLLM01000000. All data are also available at http://gryc.inra.fr.
